# Acceptability of digital health solutions and AI: qualitative interviews with perimenopausal women experiencing health inequalities

**DOI:** 10.1136/bmjinnov-2025-001406

**Published:** 2026-01-22

**Authors:** Claire Mann, Nina Kuypers, Sally Olewe-Richards, Alexandra Perry, Charmaine Daley, Poppy Taylor, Prerita Chawla, Neelam Heera

**Affiliations:** 1 CHILL (Centre for Health Innovation, Leadership and Learning), University of Nottingham—University Park Campus, Nottingham, UK; 2 Black Women in Menopause, Wirral, UK; 3 WOW-C (Women of Wisdom and Courage), Nottingham, UK; 4 De Montfort University, Leicester, England, UK; 5 Nottingham Trent University, Nottingham, England, UK; 6 This Girl Can, Nottingham, UK; 7 Biomedical Sciences, University of Nottingham—University Park Campus, Nottingham, UK; 8 Psychology, University of Nottingham—University Park Campus, Nottingham, UK; 9 Cysters, Birmingham, UK

**Keywords:** Women's Health, Gynecology, Self Care

## Abstract

**Objectives:**

Exploring the acceptability of digital health and artificial intelligence (AI) to perimenopausal women experiencing health inequalities.

**Setting:**

We recruited five community leaders representing organisations which support women who experience health inequalities, across the UK. Leaders represented ethnic minority-focused groups as well as groups representing broader disadvantage such as menopause or wellness groups based in areas of deprivation and domestic abuse support groups and were chosen to represent the experience of women in perimenopause who also experienced health inequalities. Data were collected from 84 women via five different community leaders and seven community groups in 24 qualitative datasets, which included 14 focus groups and 10 individual interviews. Inclusion criteria included experience of peri-menopause and membership within the designated group. Women who were beyond their last menstrual period were included to reflect on their experiences during peri-menopause as well as those actively experiencing symptoms. Analysis used the Theoretical Framework of Acceptability domains that influence the acceptability of health interventions.

**Results:**

Perimenopausal women expressed fear and distrust towards AI digital health interventions, particularly due to concerns about data privacy and lack of trust of representation of their racial and ethnic backgrounds. AI-driven solutions in women’s health faced scepticism due to their perceived lack of relevance and effectiveness.

**Conclusion:**

Addressing issues of representation and improving trust in AI technologies are essential to enhance trust, improve engagement and reduce digital health inequalities.

**WHAT IS ALREADY KNOWN ON THIS TOPIC**

- Previous research has shown that women, particularly those from minoritised or health-disadvantaged groups, often experience lower levels of engagement with digital health technologies due to factors such as unequal access to technology, gender biases in the design of health interventions and a lack of tailored solutions that address their specific needs.

**WHAT THIS STUDY ADDS**

- This study provides new insights into the specific factors affecting the acceptability of digital health and AI among diverse perimenopausal women, including women from racial and ethnic backgrounds and those experiencing health inequalities. Key themes identified include trust, representation, digital literacy and perceived effectiveness of these technologies.

**HOW THIS STUDY MIGHT AFFECT RESEARCH, PRACTICE OR POLICY**

- The findings highlight the need for digital health interventions to be inclusive, user-centred and culturally intelligent to ensure they are accessible and effective for perimenopausal women. Future research and policy should focus on designing digital health technologies that address the unique needs of this population, with particular attention to trust-building and ensuring data privacy.

## Introduction

 The healthcare sector is experiencing unprecedented acceleration in digital health, especially using artificial intelligence (AI).^1 2^ This rapid growth is driven in part by AI’s transformative applications across various medical domains. For instance, one study shows AI algorithms could detect lung cancer with a 95% accuracy rate, surpassing the 56% accuracy achieved by radiologists.^3^ In the UK, AI has been so successful in breast cancer screening, it is being considered for national rollout.^4^ As AI becomes more embedded in digital health, it is essential to understand how it is perceived by the public and healthcare professionals.^5^

Digital health and AI adoption and impact vary significantly across demographics. Since many women begin to experience perimenopause in their 40 s, they are likely to be classified as Generation X (GenX). This generation born between 1965 and 1980 has uniquely experienced the rapid evolution from analogue to digital technology. This group primarily utilises technology for practical applications, such as information retrieval and task efficiency.^6^ Despite their adaptability, women of this era face challenges navigating changes in the digital health landscape.^7^

The gender health gap highlights systemic disparities, where women’s health issues often receive less attention and funding.^8^ This gap is perpetuated by biases in medical research and a lack of targeted innovation in women’s health technologies.^9–12^ One digital health approach reducing the gap is FemTech, technology designed to address women’s health needs, such as period-tracking apps.^13^ Menopause tracking apps offer tools for symptom tracking, hormone monitoring and personalised health insights. One recent study^14^ suggests that menopause apps can combat epistemic injustice by equipping users with evidence to advocate for themselves in clinical settings, fostering empowerment and confidence.

Femtech is a rapid growth sector predicted to be worth $100 billion globally in 2025 and £3.5 billion in the UK by 2030.^15 16^ Some suggest that Femtech operates as a business-seeking growth and often sidelines marginal health needs in favour of scalable profit-driven solutions.^17–20^ Some perceive FemTech as contributing to ‘menowashing’, - the superficial marketing of menopause-related products that fail to address deeper health inequities.^21 22^ Health inequalities in the UK are shaped by intersecting factors such as ethnicity, poverty and domestic violence, with women from ethnic minority and low-income backgrounds facing barriers to care.^23 24^ Domestic abuse (DA) also has significant and lasting impacts on women’s physical and mental health and research emphasises that DA survivors often face barriers to accessing healthcare, which exacerbates health inequalities.^25^

Perimenopausal women who experience health inequalities are likely to face additional barriers to use of Femtech. Black and minority ethnic communities often experience lower engagement with digital health apps, partly due to mistrust and lack of culturally responsive design.^26^ Individuals with limited digital literacy or access, often those in deprived areas, are less likely to benefit from online health services, compounding exclusion.^26 27^ FemTech has been criticised for relying on data skewed towards white, affluent women and failing to address the needs of people already likely to experience health inequalities.^28 29^ Despite advances in Femtech, concerns about data privacy and security in period-tracking apps have been widely documented.^13 30^ These concerns represent further barriers to perimenopausal women in adopting such technologies.

Women are often late adopters of new health technologies. Factors contributing to this include limited access, socioeconomic barriers and a lack of targeted innovation addressing women’s specific health concerns.^31^ There is a lack of research aimed at understanding the experiences, abilities and needs of Gen X women related to digital health and AI.^32 33^ This led us to our research question: what is the acceptability of digital health and AI to perimenopausal women experiencing health inequalities?

## Methods

### Study design

The data reported here form part of a wider qualitative study exploring diverse views on menopause, digital health and AI.

We recruited a large number of participants, generating significant qualitative data. We conducted three separate analyses; the first is reported here in relation to data about digital health with findings of the second and third analyses reported elsewhere.^34 35^

In this paper, we aim to explore the acceptability of digital health and AI to perimenopausal women experiencing health inequalities.

### Patient and public involvement statement

This study was coproduced and coauthored with community leaders representing women experiencing menopause. Community leaders contributed to the design of the interview schedule, participated in data collection and analysis and writing. Their involvement ensured that the research addressed the real needs and experiences of the target population.

### Data collection

We recruited five community leaders representing organisations, which support women who experience health inequalities, across the UK. We purposively sampled female leaders to participate in a coproduction role based on their experience in supporting diverse women (and capacity) and all leaders we approached participated and none withdrew. Leaders represented ethnic minority-focused groups (black women, South Asian women, Arabic women, Angolan women, Punjabi women) as well as groups representing broader disadvantage such as menopause or wellness groups based in areas of deprivation and DA support groups. These groups were chosen to represent the experience of women in perimenopause who also experienced health inequalities. Inclusion criteria included experience of peri-menopause and membership within the designated group. Women beyond their last menstrual period were included to reflect on their experiences during peri-menopause as well as those actively experiencing symptoms. Each community leader aimed to recruit 15–20 women in 3–5 groups. The study utilised two interpreters for groups requiring language support. Data were collected through semi-structured qualitative interviews, conducted either face-to-face or online, depending on participant preference and circumstances. Where participants were unwilling or unable to attend group discussions, they were invited to individual interview.

Data were collected from 84 women via five different community leaders and seven community groups in 24 qualitative datasets comprising 14 focus groups and 10 individual interviews. All interviews were undertaken between March and May 2024. The following table 1 summarises participants:

**Table 1:** Interview participants

Community organisation (conducted by)	Participants description	Number of participants	Online or face to face	Participant number
This Girl Can (CD)	Black and Asian	10	Face to face	P1-P10
This Girl Can Arabic group (CD)	Arabic (with interpreter)	5	Face to face	P11-P15
This Girl Can Angolan group (CD)	Angolan (with interpreter)	5	Face to face	P16-P20
BWIM (NK)	Black	5	Online	P20-P25
BWIM (NK)	Black	3	Online	P26-P28
BWIM (NK)	Black	1	Online	P29
BWIM (NK)	Black	1	Online	P30
BWIM (NK)	Black	1	Online	P31
BWIM (NK)	Black	1	Online	P32
BWIM (NK)	Black	1	Online	P33
BWIM (NK)	Black	1	Online	P34
BWIM (NK)	Black	1	Online	P35
WOW-C (SOR)	DA survivors (2 White British and 1 Mixed)	3	Online	P36-P38
WOW-C (SOR)	DA survivors (3 White British)	3	Online	P39-P41
WOW-C (SOR)	DA survivors (3 White British)	3	Online	P42-P44
WOW-C (SOR)	DA survivors (2 White British/1 Black British)	3	Online	P45-P47
WOW-C (SOR)	DA survivors (2 White British/1 Pakistani)	2	Online	P48-P49
WOW-C (SOR)	DA survivors (1 White British)	1	Online	P50
Cysters (NH)	South Asian	7	Online	P51-P57
Cysters (NH)	South Asian	5	Online	P58-P62
Cysters (NH)	South Asian	5	Online	P63-P67
Switch Up (AP)	Living in deprivation (7 White British)	7	Face to face	P68-P74
Switch Up (AP)	Black and Asian	5	Online	P75-P79
Switch Up (AP)	Punjabi (with interpreter)	5	Face to face	P80-P84

Groups were audio recorded and transcribed verbatim.

Box 1 below outlines the topic guide used to stimulate group research discussions. Questions were displayed on a screen to prompt discussions:

Box 1 **- Topic guide and interview questions**

Q1) Tell me about your menopause experience

- Where are you in your menopause journey?
- When did your menopause journey start?
- How/When did you know it was the menopause?
- What symptoms have you experienced?
- How has it been for you so far?

Q2) Tell me about accessing menopause support and information

- How do you think your experience of the menopause might be different to others?
- Where do you access support and information about the menopause?
- How do you decide if you can trust information online?
- How much would you trust information shared in an app?
- Do you think there should be more/better access to support and information about the menopause? What might this look like—any suggestions?
- What do you think of/about menopause ‘role models’?

Q3) Accessing digital health

- What do you think of as digital health?
- Do you use a menopause app?
- Do you use any of these: sleep/mood tracker, exercise tracker/wearables?
- Do you use anything else you think of as digital health?
- Why did you decide to use these things?
- If you don’t use them, why not?
- What are the barriers to you using any of these?

Q4) Artificial intelligence in health

- What do you think of when you hear the term AI or ‘artificial intelligence’?
- Have you heard of any ways AI is being used in health?
- How would you feel about health recommendations made by AI based on big data?
- Any other thoughts or feelings about AI?

Q5) Your community and future work

- What are the barriers to digital health for women in your community?
- What are the barriers to accessing care and support for women in your community?
- What would help women in your community access care and support through the menopause?
- What kind of support would your community value most for the menopause?

We were interested in women’s broadest experiences of menopause to contextualise their experiences of digital health. The data reported in this paper focus on the final three questions on the topic guide and are priori focused, aiming to understand the acceptability of digital health to perimenopausal women. Data from earlier questions were independently subjected to an emergent thematic analysis and elsewhere we report on the broader findings related to women’s experiences of perimenopause.^36^ We also conducted an independent analysis of data relating only to DA survivors^37^ and found these data to broadly correlate with the wider data collected and no notable contradictions.

### Data analysis

Data were coded using a priori framework derived from the Theoretical Framework of Acceptability (TFA) key domains that influence the acceptability of interventions: affective attitude, burden, ethicality, intervention coherence and opportunity costs, perceived effectiveness and self-efficacy.^38^ This framework was selected to provide focus to answer the research question about the acceptability of digital health and AI to perimenopausal women experiencing health inequalities. Data were also subjected to emergent analysis, which is reported elsewhere. All data were meaningfully captured within the predefined framework, and each category was sufficiently populated, indicating analytic completeness and a strong fit between the data and the framework.

Initial coding involved two student researchers (PT and PC) coding 50% of the data each, while the third, a more experienced researcher (CM), coded 100% of the data. The final coding results were refined into a summary table that underpinned each subtheme within the TFA domains. Intercoder reliability was assessed by comparing the coding results, and any discrepancies were resolved through iterative discussion within the coproduction group. Supporting data is in online supplemental Data S1.

## Results

### Affective domain

The affective domain represents how users feel about an intervention.

Participants expressed a range of feelings, some positive but many more negative. Many expressed a lack of understanding of digital health interventions or AI with some suggesting their lack of understanding or interest was because of their age and others attributing it to a lack of clear public information. Participants from black, Arabic and Asian populations reported that interventions including digital health devices and in particular digital health videos targeted an upper middle class white audience. Participants in all groups expressed fear of digital interventions using AI. Many participants also reported a wider lack of trust in health systems, which extended into digital health. Several participants, in particular, Asian women and those with experience of DA, had concerns about data privacy. There were some positive views with some women feeling motivated and empowered to control and improve their health using digital health devices or apps, and some women were optimistic about the potential for AI; however, these were minority voices across the groups. Overall, participants represented a range of mixed feelings highlighting both fear of but potential for digital health in particular with AI.

People are equally like scared and thrilled by it. I think it’s clever, but I'm also terrified by it. (Participant 54, Asian woman)

### Burden

The burden domain represents the amount of effort perceived in order to participate in using a digital health intervention.

Participants in every group said preferred interventions that were simple to use and intuitive and wherever possible required only passive interactions, such as wearables, which recorded data automatically being preferred over apps requiring the user to manually enter data. Many participants felt they were not digitally literate enough to maximise the benefit from use of digital. Most participants discussed the many competing priorities that might prevent them from using a digital health intervention and suggested that due to these competing priorities, they did not have the discipline required to maintain use of an intervention to reap the maximum benefit. Participants evaluated the efforts of using a digital health intervention against the perceived reward and for many this represented a deficit that leads to a lack of engagement over time.

But after a while I found it a bit overwhelming having to, you know, put in all your info. I mean, I loved it because it gave you, like, an analysis of the end and then at least I can check to make sure that I was, you know, getting a balance. But yeah, I stopped doing that after a while. (Participant 28, Black woman)

### Ethicality

The ethicality domain considers whether the intervention is a good fit with the value system of the stakeholder.

Participants wanted to feel that digital health interventions were created for people like them and many suggested that they were more acceptable if they seemed to be representative of the way individuals recognised themselves, including race, ethnicity, disability, gender, age and physical attributes like shape and size. This was particularly relevant to digital health videos where participants rarely saw themselves represented but also in the ownership of solutions by people they did not see as representative or understanding of their background and needs. A small number of participants recognised the importance of big data and AI for the greater good. However, overall, most participants showed a lack of trust of AI recognising their individual needs or data being used to benefit them.

I think what happens is with all of this, programming it is from a very euro male perspective. Yeah. So, I don't trust it. I don't. I wouldn't trust it at all. (Participant 54, Asian woman)

### Intervention coherence

The intervention coherence domain considers whether stakeholders have a good understanding of how the intervention works.

Participants in most groups watched health videos and considered the source and site of the information as important. Most groups discussed wearables and suggested that live tracking of data was a good technique, which was well understood as an opportunity to engage with, monitor and improve various aspects of health. When discussing AI and the use of data, participants ranged from having little to no understanding of the practical application of AI in digital health through to having a good appreciation of the benefits of collecting big data for personalised medicine. Most participants understood the benefits of digital health tracking.

I think it looks great in terms of getting to know yourself during this time to see if there’s any patterns. And I think especially with brain fog and things like that, having that sort of accountability there online that you can look and get to know yourself is good. (Participant 73, Asian woman)

### Opportunity cost

The opportunity cost domain considers what users have to forego in order to engage with the intervention.

Many women referenced the financial costs of purchasing digital health devices or apps and expressed a need to evaluate paying for something non-essential. Some groups were aware of ‘meno-washing’.

And you don't want to pay for something you don't have a guarantee if it’s free because you don't want to pay for something at the end of the day, it’s not gonna work well. (Participant 28, black woman)

Participants across all groups expressed concerns about finances, especially those women who had experienced DA or poverty. Participants in all groups discussed the cost of their time spent on digital health that could be spent on other priorities, and some women explained that time was required to learn how to use new devices or form new healthy routines as well as for day-to-day use. Some women had concerns about the potential societal cost of AI, even going so far as to draw comparisons with the portrayal of dystopia they have witnessed in media.

### Perceived effectiveness

The perceived effectiveness domain considers how effective users perceive an intervention to be. This included examples of users who perceived interventions to have low effectiveness.

Participants suggested interventions were ineffective if they lacked clear benefit or did not function as perceived. While many examples given of ineffective interventions were provided, fewer examples of effective ones were mentioned. The most cited example of a digital health intervention perceived as effective was a step counter (mostly used on phones).

Participants discussed the ways in which they perceived AI to be more or less effective than a human at health tasks. AI was considered by some to be less effective than a human at interpersonal interactions, but some considered AI more effective than humans in using big data to understand health problems, and also due to limited resources for clinical support and the taboo nature of menopause discussions for many women. One participant suggested that humans and AI could work together for the best outcomes.

At the moment, if you have something wrong with you, sometimes it takes the doctors and nurses you know quite a while to just figure out what’s wrong with you. With AI, I think that that would just help to speed up, you know, then finding what the problem is. You know to actually diagnose the problem. So, I think AI will be very helpful in that area. (Participant 29, black woman)

### Self-efficacy

The perceived effectiveness domain considers the confidence of participants to use the digital health intervention.

Some participants expressed low confidence due to a lack of motivation to change habits and fear or perceived lack of technical ability. However, many users showed strong self-efficacy in trying digital health interventions, especially when recommended by trusted individuals. Many users identified that training or advice from professionals into how to best engage with digital health interventions created strong self-efficacy.

I need that 100%. I need somebody to tell me what to do and I am one of those people I will just do as I'm told. (Participant 47, Domestic abuse survivor)

These results overall demonstrate the range of acceptability of digital health from interventions, which were not acceptable to some users to those which were fully embraced. Furthermore, we identified some key factors affecting the acceptability of digital health interventions, particularly those likely to experience health inequalities.

## Discussion

Women facing health inequalities often being late adopters of new digital health innovations have the most to gain but are often the least likely to benefit.^39^ We identified factors that understanding, trust and representation are key factors shaping acceptability, aligning with previous research that digital health adoption is influenced by sociodemographic factors, digital literacy and systemic biases in healthcare.^39 40^

### Factors affecting acceptability of digital health interventions

Acceptability varies depending on the type of digital health intervention. Wearable devices such as step counters were widely accepted due to their ease of use and tangible benefits, whereas AI-driven solutions faced greater scepticism, particularly due to a lack of understanding.^41^ AI was often perceived as disconnected from personal experiences, making trust a key barrier to adoption.^36^ Many participants expressed fears about AI-based interventions, particularly around data privacy, algorithmic bias and the risk of misinformation.^37^ These concerns highlight broader ethical issues in digital health, including data security and representation of diverse people and communities in AI training datasets.^42^

Participants described the cost, both financial and time, of engaging with digital health interventions and the competing priorities that limit their ability to adopt new technologies.^39^ This supports existing research indicating that digital health solutions must be intuitive, require minimal effort and provide clear value to users to ensure long-term engagement.^40^

### Maximising the effectiveness of digital health interventions

This study provides insights into how digital health interventions can be made more acceptable to perimenopausal women experiencing health inequalities.

Trust plays a crucial role in adoption. Previous research has shown that AI and digital health interventions must be transparent and designed with user needs at the forefront to improve engagement.^43^ Participants highlighted that personalised recommendations and culturally inclusive content could increase their willingness to engage with digital health technologies.^44^

Perceived effectiveness also influenced adoption. Women were more likely to engage with technologies that provided immediate and clear benefits, such as real-time health tracking.^45^ By contrast, there was a lack perception of the effectiveness of AI-driven applications, particularly due to concerns about their accuracy and applicability to women’s health needs.^46^ Prior research suggests that a user-centred approach, involving codesign with target populations, is essential for improving digital health uptake.^47^ How well participants understand technology is a critical factor. Many participants struggled to define AI’s role in healthcare and were uncertain about how digital health tools function.^48^ Addressing these gaps through education, user-friendly design and clinician support could significantly improve the acceptability of digital health innovations.^49^

### Strengths and limitations

The sample primarily included women engaged with community groups, which may not fully capture the perspectives of those less socially connected. The focus on menopause-related experiences means that the findings may not extend to other health concerns affecting this demographic.

Coproduction is the key strength of this study, ensuring that community voices were embedded throughout data collection and analysis. The diverse sample, including women from various racial and ethnic backgrounds and experiences of health inequalities, enhances the study’s relevance. The use of the TFA provides a structured lens through which to interpret findings, offering clear insights into the barriers and facilitators of digital health adoption.

## Conclusion

This study highlights factors influencing the acceptability of digital health and AI among perimenopausal women, particularly those experiencing health inequalities. While digital health interventions hold promise for improving health outcomes, adoption is hindered by digital literacy, perceived effectiveness and trust. AI is not well understood or trusted; addressing concerns about AI and data privacy through clear communication and regulation could enhance trust. Future research should focus on developing and evaluating accessible, effective and culturally intelligent FemTech to meet the needs of all perimenopausal women, especially those experiencing health inequalities who face the most barriers. By tailoring digital health innovations to this population, we can work towards reducing health inequalities and improving long-term health outcomes for women.

## Supplementary material

10.1136/bmjinnov-2025-001406 online supplemental file 1

## Data Availability

Data are available upon reasonable request.
