# Supplementary material for: Acceptability of digital health solutions and AI: qualitative interviews with perimenopausal women experiencing health inequalities
Source: BMJ Innov. 2026 Jan 22;12(3):e001406. doi: 10.1136/bmjinnov-2025-001406 (PMC13479213; doi:10.1136/bmjinnov-2025-001406)
Supplement: Supplementary file 1 [file bmjinnov-12-3-s001.docx]

| **Domain** | **Theme** | **Comment / Quotes** |
| --- | --- | --- |
| **Affective Domain** | **Understanding**  [Digital health(DH)]  [AI] | Just not being aware of it, and maybe that goes with age and the rest of it (P7)  I feel uncomfortable about it [AI], honestly, but that's probably just because it's new and I am just losing my interest in new things. I feel so old. But yeah, I'm nervous (P61) |
|  | **Representation**  [Devices]  [Digital health videos] | Yeah, I think it's a lack of my story being heard. It's also [the] lack of my story being told in public spaces. And also they [digital health videos] just come across as only certain women have value (P28)  Digital health feels very bougie and upper middle class and you know people running in the park with their Fitbit. I feel a bit like that, and I would not be classified as low socioeconomic income (P55)  Like the barriers to using them [DH / Videos] is because they're not tailored to us. You don't know how much some of the advice all the articles are actually reflecting what you were going through or what we’re going through. (P64) |
|  | **Fear**  [AI] | I'm just scared you know. I think it's a great thing, but I am scared they're gonna take over the world. (P3)  For me it's so scary. (P13)  There are lots of ‘ifs and buts’ for AI. I do have reservations about the AI (P55)  I think is dangerous. (P56)  The word or the phrasing of ‘AI’ makes it scarier. But maybe it could just be [said] in a way that's a bit softer and doesn't make you think of robots and stuff. (P62)  It's a very dangerous place we're in (P67) |
|  | **Trust**  [Health systems]  [Data] | In a way it’s trust, it’s about experiences of inequality, if they’ve experienced that from real humans, they're not going to trust an app where they're not talking to someone. So that's a major barrier. They aren't going to use it if they already don't trust the healthcare system. (P2)  I get really nervous about all the kind of settings and the stuff that they read from your phone (P31)  I'm not the sort of person that will share my data with everyone. I'm not. I just won't. (P34)  The only thing that always scares me is like it's, it sounds ridiculous, but it's always like the use of the data. And I think this is like from my ex, .. information is power and anything he can use against you. So, I'm always like very much like ‘what do they want to know and why?’ And I want to know that it's not gonna be, somehow connectable with me. (P42) |
|  | **Positivity**  [AI]  [Digital Health]  [Data] | I think a positive of social media and AI [is] that you can reach more people. (P29)  I love monitoring and sort of aiming to beat .. how many steps I did the previous day (P29)  Yeah, as long as it’s, there's no personal details, I won't mind that at all. I think that's really good because we're all logging on to be helping each other (P52) |
|  | **Mixed Feelings**  [AI]  [Data] | People are equally like scared and thrilled by it. I think it's clever, but I'm also terrified by it. (P55) |
| **Burden** | **Simplicity**  [DH]  [Apps]  [Period tracker] | Should be simple, easy to do it, to use [it] (P15)  Like multiple models and not knowing whether it's compatible with all the other tech that you already got and whether what, where it comes from and how to get it and what to do with it (P54)  I don't think I'd use [a period tracking app] just because I could probably put it in a journal. (P55) |
|  | **Digital literacy**  [Apps] | [Using an app] could be difficult for the older generation. Like I'm on the phone, they don't know how to get access on the phone, they [are] still going to struggle. And then if you go on it... if there's 10 questions, how they gonna get onto it, how they gonna download the things we still have to do it for the right way. Yeah, we'll have to download it for them and do it, and it's still gonna be difficult (P77) |
|  | **Competing priorities**  [Digital Health]  [Apps] | I also feel sometimes overwhelmed by the sheer amount of things on my phone and the sheer things dinging and buzzing and beeping. (P62)  You know, finding the time to use it as well. (P75)  Yeah, it's too much sometimes, isn't it? Just an extra thing. (P77) |
|  | **Discipline**  [Digital Health]  [Apps] | I just think with the apps it's a lot. It takes a lot of discipline. (P55)  I just know that I’d been kind of, I'll be all gung-ho about it for like 5 days and then it would just peter off and I'd never bother doing it again. (P48) |
|  | **Trust**  [AI]  [Data] | It's not everybody that wants to use it again because of our conspiracy theory of saying it’s going to take over the world. (P22)  I think also in marginalised communities what we're seeing more and more is just this lack of trust of the mainstream and I don't know if everybody, even if they're confident with tech, [they] wouldn’t necessarily want to share their data. (P32) |
|  | **Reward**  [Apps] | I have the [menopause] app on my phone, but I'll be honest with you, I don't use it. I've looked at it and you know, it doesn't really help me really, so I don't really use the app at all (P28)  That is just literally like a diary, and you can just do that yourself on your own calendar or notes. So, it doesn't really add any value (P27) |
| **Ethicality** | **Representation (Ethnicity)**  [AI]  [Digital Health]  [Data] | If it's OK for all women but black women are different, brown women different. We're all different. So, if it is just based on a white woman then it's like, yeah, you're gonna help me. Yeah. I get your helping me, but you're not helping me properly. (P3)  I think my experience might be different, yes because of my race and therefore my culture. I'm trying to think about Islam specifically. So, if we think about religions in general, particularly you know South Asian communities generally quite religious, or spiritual, you know, even if you don't practice your religion to the tea.  I think there's something about, you know, how do we have spaces where we look at the role of spirituality and God in the menopause journey? Yeah. And that's something that I think is just like, you know, for the Western idea would be like ‘Whoa, that's weird.’ (P55) |
|  | **Representation (other)** | I'm not being funny. I'm like 48 nearly and this is wrong really, but it's a 19-year-old male who was gonna sit and tell me about myself, I don't want that- you know what [do you know about] me? (P4) |
|  | **Trust (Ethnicity)**  [AI]  [Digital Health]  [Data] | It is a bit western-centric, so [there is] not enough research on people of colour to actually draw from in AI. (P7)  I don't know if I would trust AI based on big data because I know a lot of these things are based on, you know, like white people of a certain, you know, demographic and would it fit? (P62)  And we already know that AI has been proven to be, [that it] does discriminate. So, for someone like all of us in the room, where is it going? It's unlikely to benefit us directly. And how do we train that out? I just it feels so unregulated. I wouldn't feel comfortable using an AI based health app. (P63) |
|  | **Trust (Authorship)** [Digital Health]  [Data] | So how do I decide if I can trust information? I always look at research evidence, sources, perspectives. Who's done the research? How much would I trust information shared in an app… I think that depends on how the app was created. (P55) |
|  | **Trust (Fakes)**  [AI] | Look at those rappers like singing rap songs, and they've had to come out and say that wasn't me. It's AI like, people are taking your images. For me, it's scary as hell. It's so real. Like, yeah. How do you then trust the CCTV if a crime is committed and someone is committing a crime, how do you trust CCTV? If AI can manipulate everything, I think it's great that you can ask it a question and it answers it. I certainly wouldn’t want a robot predicting my health or telling me what’s wrong with me (P3) |
|  | **Greater good**  [AI]  [Data] | Look at the wide picture. It's for the benefits of other people to know, to understand, to share the experience (P15)  I think me personally, I prefer, I'd be happy with having my data used to help other people because of other people's data would be then you to help me, which would be good. (P29) |
| **Intervention coherence** | **Digital health - active**  **[Wearables / tracking]** | An online tracker that tracks your health or that you can monitor it, but we can also change it like the calorie counter, to adjust to yourself and personalise it then. (P4)  You can get this app and it takes a photo of your body. Yeah. And then it creates a fitness trainer based on your body and it works out, I don't know how it works out your BMI well I suppose you put the numbers in. And then… it tailors the exercises for you, then you're getting rid of a fitness trainer, cause that's what fitness trainer does. (P28)  I like to know how far I run, how fast I have run. I'd like to know what to exercises I’m doing, how long I've done it for, how many calories I've burned and I think for me that's a good motivation. And it's also there's a good motivation to see when I'm improving week on week, month on month just gives you that feedback, isn't it? (P30) |
|  | **Digital health - passive**  **[videos]** | I do YouTube fitness videos, so is that digital health? I don't know. Or is that more like consuming content? It's an interesting one. (P55) |
|  | **Strong understanding**  **[AI]** | That'll be really good that artificial intelligence is also able to be diversified in their responses to people with different ethnic groups (P17)  I mean AI in general I think, is potentially very, very useful, but as we know AI is only ever as good as the people that feed into it. (P32)  I know that you know the more information you put into it, the more it learns and then the more it can make, you know, probable suggestions to you as to what it might be. Because statistic, you know statistics and numbers and all that clever stuff. (P35)  Nothing can replace that personal touch, that face to face contact. But it could, if it is able to harness the right kind of research and knowledge that is out there accurately… Say you had some kind of consultation.. with a polite bot. You know you can put all your details in there .. your racial background, your religion and everything else body-wise and your symptoms, whatever you're feeling. It could pull from so many relevant and reliable resource and sort of resources to maybe give you a good path to begin on almost or at least some kind of an idea of where you might be within your journey at that time would be of menopause or perimenopause, or on anything… I don't think it could ever, and it shouldn't ever take over everything. However, I think it can be a really good, especially nowadays where it's so hard to get heard, be heard by anybody sometimes (P64) |
|  | **Weak understanding**  **[AI]** | I suppose because it's artificial, but it's really there's a person behind it, isn't it? Somebody will had fed it the data, certain data profiles and things, but it cannot really 100% reflect every single person’s Uh profile, so it would, It's more like an estimate, so. How would how would it know the difference between me and you? For example, in terms of recommendations, how can it give me personal personalized Recommendation? I don't (P30)  And this AI that they're talking about saying in years to come, the robots will be doing operation on people is all artificial intelligent and taking people's data. Because I always saw on the TV when the robots..like they put human brain on them, talking and [it] is like they are going to take over the world (P56) |
| **Opportunity cost** | **Financial costs** | I think in the current climate, when you know, everybody's feeling it at the moment, you have to kind of decide whether these apps are useful enough for you to spare a tenner a month. So, the biggest barrier for me in using any of these is how much they cost, how much of a recurring cost they're going to be. (P35)  It isn't a necessary cost on top of all the other maintenance that you have for your health (P63)  And there's a bit of me that's thinking [that] I shouldn't have to fork out for this (P65)  I struggle at the moment with financial abuse. And so if all your money is going into all the bills and everything…so many women have this situation, I think ‘try this’ … but you don't even have that extra cash anyway. (P39) |
|  | **Time / Effort costs** | It's a chore in itself to put things into the app. But then if you don't put them into the app, they're not that helpful. (P58)  There's so many calendars. There's so many meetings. There's so many books, subgroups to keep an eye on. So there's so much already going and on top of it, I'll put something else on it..And same with menopause. There's so many health apps out there, and as soon as [you have to] put your information in like the lady said this it just it just gets too much (P66)  I started factoring in time in the day to actually start, you know, logging things. On the on the train, at the end of the day, at the beginning, lunchtime and so on. Yeah, it's a bit of a lifestyle change to stop and make an entry and so on. (P32)  So you know [if] something's too long, I just lose interest. You know, everybody's busy aren't they? (P52) |
|  | **Societal costs**  **[AI]** | I think it takes away if we're gonna rely on sort of AI for our health and if it turns into AI doctors for example, that takes away [the chance for] doctors to learn more about the conditions that we're going through. (P63) |
| **Perceived Effectiveness** | **Ineffective**  **[Doesn’t function well]** | Like I've got a Fitbit and it tracks your periods, but mine could never track because it was lasting for so long as well, mine, It was so abnormal but like for menopause tracker, it tried to track but it couldn’t track. So [I was bleeding] not like normal women would probably have, every four weeks and last five days. Yeah, it tracked that quite easily. Until you kept on [bleeding] four days. But my daughters got a period tracker works perfectly, but she's young and, you know, she has normal periods, but with mine it just couldn’t track them. (P2)  So, I downloaded something from Facebook that was telling you could monitor your blood pressure and your heart. It was telling me things were normal, but I went to the doctor's today and it was high..(P10)  Alexa very often gets things wrong. You know, you ask for things and it offers you other stuff. So I suppose you have to be conscious of that, don't you? Because, say is incorrect, but it still sounds convincing. Then that person can make a decision, presuming that the information is correct. You know, so that's that, I suppose when you think about it, that's quite worrying. And you don't really know how to get around that, do you? (P27) |
|  | **Ineffective**  **[Not clear benefit]** | If someone explains to them how the app will actually help them with their symptoms, then that may give them an incentive to [use it] (P17)  I remember looking at it and thinking I don't understand what this is gonna do for me. Why am I tracking my symptoms. What's the point? I know what I'm going through. I don't really need an app, right? (P67) |
|  | **AI ineffective**  **[in relation to humans]** | Also, if someone writes “I’m sad,” versus you looking at someone and seeing that they’re sad but they haven't said the word. AI can't pick up someone’s emotions. (P2)  Suppose if it’s like skin and what type of dermatology are they talking about? Our skin type is different, and we require different, not mainstream creams. So, it would need to be for black and brown skins (P3)  [If you] Google … your symptoms, it used to always give you horrible symptoms that you're almost gonna die and .. so I don't trust. And they don't understand. They don't have feelings. like in, in the real world. Like a GP would know what you're saying and what's wrong with you but for artificial intelligence [that] feels too difficult. You will die. (P13)  I think that this is a machine, and it can break down at any point. This Internet breaks down (P25)  The thing is with AI is, in theory it should cannibalise itself because there's only so much it can process and that's the same things going over and over and churning for a sort of content like blogs and things. Yeah, it it's not going to have as much longevity as people think it's going to have. (P55)  All these conditions are so gravely misunderstood. by doctors right now, how is the computer system going to understand that well enough to give me advice on my health? (P63) |
|  | **AI effective**  **[in relation to humans]** | I think if you told if you told the AI about your menopausal symptoms, I think you'd probably get a better response than you can from a dude that may or may not have done a 20-minute call. Do you know what I mean? (P3)  Why sit and describe it to a 20-year-old boy when you can just describe it to an app? (P4)  GPs aren't that great and also you can use an AI and it's accurate it you get rid of the kind of the embarrassment element. (P28)  if you have something wrong with you, sometimes it takes the doctors and nurses you know quite a while to just figure out what's wrong with you. With AI, I think that that would just help to speed up, you know, then finding what the problem is. You know to to actually diagnose the problem. So, I think AI will be very helpful in in that area. (P29)  I think if certain medical professionals could just get out of their own bottoms and see that an AI or something like this could be beneficial for them in terms of time saving. Yeah, that actually could keep us all away from, you know, not being seen for weeks and weeks and weeks and weeks. I think it's definitely worth exploring and it is already being used…they do it for diagnostics, right? They do it already. (P67) |
|  | **Effective**  **[Step counters]** | I use them just to check my steps on daily basis. Should be at least 10,000. So, if I'm struggling, I just do some work inside the house. And [I use] the app on my mobile, I don't use the watch. (P15)  She always loved and enjoyed walking but also was always walking to work and she wanted to know how much she was getting out of it. So having an app like that would allow her to see if she did 10 to 12,000 steps a day and that she wants to make sure that every day she did the same amount so that she kept that level of activity. (P17) |
|  | **Effective**  **[other]** | I've got a Fitbit as well and I use that hell of a lot. So, it monitors my sleep and I find that really good. So, there were times when I didn't used to get enough sleep but with the Fitbit, at least I can monitor that. (P29) |
| **Self Efficacy** | **Low confidence (fear)** | I was going to say it's just a bit overwhelming having like different apps for different things. (P28) |
|  | **Low confidence (lack of technical ability/access)**  **[Digitial Health]** | Could be difficult for the older generation. Like I'm on the phone [but] they don't know how to get access of the phone, they [are] still going to struggle. And then if you go on it and .. if there's 10 questions, how they gonna get onto it, how they gonna download the things we still have to do it for the right way? Yeah, we'll have to download it for them and do it and it's still gonna be difficult (P77)  So I didn't understand how to use it.’ Maybe that's my age and my generation [but] I just didn't understand. (P67)  My first problem is with this artificial intelligence thing, how many people will have even the recommended material like a phone, an iPad or something, to be able to access? And even if they have, how many will be able to do it to assess it to even really log on to it properly and do the right thing? (P25) |
|  | **Willing to try**  **[Digital health]** | There wouldn't be a reason why I wouldn't use it as long as I got in the flow with it (P52) |
|  | **Wiling to be led by trusted others**  **[Digital health]** | It's around education of digit of using digital apps. Not everyone uses apps. I mean, I know everyone has access to them. I mean, I hardly ever use QR codes. Everyone else seems to, but I just don't hardly ever use them. So, there's an uncomfortableness, I suppose, in using digital health as a habit. (P28)  What about AI? I need it explained to me like as a sort of 13-year-old science level other than that, I just I get lost. (P54)  Things like accessible workshops and you know, things that if you've got an accessible workshop where people can go to it and you can explain how to use [digital health apps]. (P70) |
